# GTL-1, a Calcium Activated TRPM Channel, Enhances Nociception

**DOI:** 10.3389/fphar.2019.01567

**Published:** 2020-01-17

**Authors:** Emiliano Cohen, Rakesh Kumar, Tal Zinger, Avi Priel, Millet Treinin

**Affiliations:** ^1^ Department of Medical Neurobiology, Hadassah-Medical School, Jerusalem, Israel; ^2^ Faculty of Medicine, School of Pharmacy, The Institute for Drug Research, Hebrew University, Jerusalem, Israel

**Keywords:** TRPM, *C. elegans*, nociceptors, calcium, behavior

## Abstract

*C. elegans* PVD neurons are conserved for morphology, function and molecular determinants with mammalian polymodal nociceptors. Functions of polymodal nociceptors require activities of multiple ion channels and receptors including members of the TRP family. GTL-1, a member of the TRPM subclass of TRP channels, was previously shown to amplify PVD-mediated responses to optogenetic stimuli. Here we characterize effects of GTL-1 on PVD-mediated behavioral responses to noxious stimuli. We show that GTL-1 is required within PVD for the immediate and enduring response to thermal (cold) stimuli. But, find no significant reduction in percent animals responding to single or to repeated noxious mechanical stimuli. Nevertheless, PVD specific knockdown of *gtl-1*expression reduces the magnitude of responses to noxious mechanical stimuli. To understand GTL-1’s mechanism of action we expressed it in HEK293 cells. Our results show GTL-1-dependent currents induced by activation of a Gαq-coupled Designer Receptor Exclusively Activated by Designer Drugs (DREADD). In addition, using excised patches we show that GTL-1 can be activated by internal calcium. Our results are consistent with indirect, calcium dependent, activation of GTL-1 by noxious stimuli. This mechanism explains the GTL-1-dependent amplification of responses to multiple stimuli optogenetic and sensory in PVD.

## Introduction

Responding appropriately to noxious, potentially injurious or injurious signals is essential for survival. This response requires polymodal nociceptors, multi dendritic sensory neurons innervating the skin or internal organs and responding to high threshold thermal and mechanical stimuli and to noxious chemicals (Woolf and Ma, 2007; Smith and Lewin, 2009). *C. elegans* PVD neurons are multi dendritic neurons, conserved for structure, function, and molecular determinants with mammalian polymodal nociceptors (Chatzigeorgiou et al., 2010; Albeg et al., 2011). These neurons respond to high threshold mechanical stimuli and to cold temperatures, responses mediated, respectively, by the DEG/ENaC channel subunits, MEC-10 and DEGT-1, and the TRPA channel, TRPA-1 (Chatzigeorgiou et al., 2010).

Transcriptomic analysis of PVD neurons identified multiple ion channel subunits likely to express in these neurons (Smith et al., 2010). Several of these channel subunits were examined for their effects on the behavioral response to optogenetic activation of PVD (Husson et al., 2012); this study identified GTL-1 as an ion channel needed within PVD for amplification of the behavioral response to optogenetic stimuli. GTL-1, Gon-Two Like protein, belonging to the TRPM subfamily of the TRP superfamily was previously shown to function together with GON-2 in maintaining Mg^2+^ homeostasis in the *C. elegans* intestine, as well as in controlling IP_3_-dependent Ca^2+^ oscillations to control defecation (Teramoto et al., 2005; Kwan et al., 2008; Xing et al., 2008; Xing and Strange, 2010). However, roles of GTL-1 in responses to PVD-mediated, physiological, noxious stimuli have not been studied.

Members of the TRP superfamily play key roles in nociception (Clapham, 2003; Dhaka et al., 2006; Julius, 2013). TRP proteins form nonspecific cation channels shown to respond to noxious heat, noxious cold, pH, and mechanical stimuli. For example, TRPV1, also known as the capsaicin receptor, is expressed in mammalian nociceptors and is important for mediating responses to heat, acid and vanilloid toxins (Caterina and Julius, 1999; Caterina et al., 2000); TRPM3 functions as a noxious heat sensor (Vriens et al., 2011); and TRPM8 functions as a cold sensor (Dhaka et al., 2007). Functions of TRPs in polymodal nociceptors are not limited to nociception as they were also shown to modulate nociceptor activity. For example, in the *Drosophila* DA-IV polymodal nociceptors, TRP channels functioning downstream to hedgehog signaling sensitize responses to noxious stimuli (Babcock et al., 2009). Indeed, several TRPs, including TRPM channels, were shown to be activated by intracellular Ca^2+^, suggesting that in certain cases they function downstream to Gαq-coupled GPCRs, other TRP channels, or voltage activated calcium channels (Launay et al., 2002; Zurborg et al., 2007).

Here, we examine whether, GTL-1-dependent amplification of optogenetic stimuli (Husson et al., 2012) represents a physiological role for this channel in enhancing responses to noxious stimuli. For this, we examined effects of loss-of-function mutation or PVD-specific knockdown of this gene on PVD-dependent responses to thermal and mechanical stimuli. Results of this analysis show defects in these responses, supporting a role for GTL-1 in PVD-mediated responses. In addition, we expressed GTL-1 in HEK293 cells to show that, like other TRPM channels, it is activated by internal calcium and can function downstream to Gαq-coupled GPCRs.

## Materials and Methods

### Strains

The wild type is N2. The *gtl-1(ok375)* mutation is a loss-of-function mutation due to a deletion of 2.7Kb encoding for all the transmembrane domains of GTL-1, the strain carrying this mutation is referred to as *gtl-1(lf).* The *trpa-1(ok999)* mutation encodes a loss-of-function mutation of *trpa-1* due to a deletion of 1.3Kb, encoding for most of the intracellular N-terminal domain. The *mec-4(1611)* mutation is a gain-of-function mutation leading to degeneration of the six touch receptor neurons (AVM, ALM, PVM, PLM). This strain was crossed with the *gtl-1(lf)* strain for the high threshold mechanical stimulation assay, and also served as a control in that assay. The integrated *ser-2prom3::deg-3(N293I)* transgene which eliminates PVDs via degeneration (-P animals), was previously described in (Albeg et al., 2011).

### Plasmids

To knockdown expression of *gtl-1* in PVD we used the method developed in (Esposito et al., 2007) for expressing double stranded RNA of genes of interest in specific cells. Specifically, coding inserts of *gtl-1* were amplified from the *C. elegans* RNAi library (Source BioScience, clone IV-6A13,(Kamath et al., 2003)) using primers L4440F (5’ CGACGGTATCGATAAGCTTGAT-’3) and L4440R (‘5- CGACGGTATCGATAAGCTTGAT-’3) each containing a HindIII site. The resulting 1.1 Kb fragment was cloned in both orientations downstream to a 1.7 Kb *ser-2prom3* promoter (expressing in PVD and OLL (Tsalik et al., 2003)) in the pBluescript SKII(−) HindIII site. The plasmids expressing both orientations were injected at 50 ng/μl each with a *ser-2prom3::*GFP plasmid as a marker into wild-type N2 animals.

A full length cDNA of *gtl-*1 cloned into pGEM-T was a kind gift from Howard Baylis (University of Cambridge). For expression of GTL-1 in HEK293 cells, a 5kb KpnI-BamH1 cDNA fragment containing *gtl-1* was cloned into pCDNA4/TO.

### Behavioral Analysis

#### Response to Cold Temperature Following Prolonged Exposure

The assay was performed as previously described (Albeg et al., 2011; Cohen et al., 2014). In brief, for movement analysis animals were transferred as L4 larvae to fresh plates and grown overnight to adulthood at 20°C. Prior to locomotion analysis single adults were transferred to a fresh NGM plate pre-equilibrated at 20°C or 15°C and having a thin layer of OP50 bacteria as food (overnight growth) and allowed to acclimate for 10 min at 20°C or 15°C. Movement of each animal was then recorded at a 25× magnification and at a rate of 10 frames per second. Animals were recorded for 60 sec or until they moved out of the frame. These movies were analyzed using software developed for this purpose [(Albeg et al., 2011), detailed description of this software is provided in http://www.cs.huji.ac.il/~feit/worms/user-manual.pdf] .

#### Immediate Response to Cold

For rapid and precise temperature control of a thin agar surface on which the animal’s movement is recorded we developed the following experimental setup: one animal was transferred to a small piece of NGM agar. To keep the animal within the imaging field it was placed on a dot (approximately 2mm in diameter) of bacteria. The agar was placed on a cover slip and put on top of a thermoelectric plate (peltier, European Thermodynamics ET-127-20-25) and a thermocouple unit (National Instruments TC01) was inserted in the agar. The peltier unit was connected to a programmable power supply unit (Thurlby Thandar Instruments PL-155-P) and a digital microscope (DinoLite Pro AM413T). The power supply unit, the thermocouple unit and the microscope were connected to a PC running a Matlab program that can record the movement of the worm, receive the information from the thermocouple and adjust the voltage of the power supply, thus, maintaining or changing the incubation temperature.

The automated protocol to test the effects of cooling on animal behavior was as follows. First the temperature of the agar was equilibrated to 20°C. 10 min after this temperature was achieved and stabilized, the temperature was dropped to 15°C or maintained at 20°C for control purposes. The animal remained at this temperature for 10 min, followed by a return of the temperature to 20°C. Recording and analysis of control and temperature drop experiments were randomized and blind to the animal’s genotype. Using this imaging setup we were only able to examine reversal frequency (direction changes from forward to backward movement) and this analysis was done manually. Reversals were counted for a period of 30 sec just before the temperature drop, immediately after the temperature reached 15°C, after 10 min at 15°C, immediately after the temperature returned to 20°C, or at the same time frames for control animals maintained at 20°C,.

#### Mechanical Stimuli

For response to high threshold mechanical stimulus young adult (picked as L4 to fresh plates, 10 animals per plate, and grown at 20°C overnight) *mec-4(e1611)* and *mec-4(e1611); gtl-1(ok375)* animals were examined for their response to prodding with a platinum wire pick. Animals reversing or increasing their forward speed were considered as having responded (Way and Chalfie, 1989). To test adaptation to high threshold mechanical stimulus, young adults (picked as L4 to fresh plates and grown at 20°C overnight) N2, *gtl-1(ok375)*, and -PVD animals were examined for their response to prodding with a platinum wire pick every 10 sec (Husson et al., 2012).

Prolonged response to noxious mechanical stimulus (transfer with a wire pick) was examined as described in (Cohen et al., 2012). Briefly, animals were grown at 20°C on standard nematode growth media (NGM) plates seeded with the *E. coli* strain OP50. For each assay, L4 animals were transferred for overnight growth to fresh NGM plates and examined as young adults. At the start of each experiment a single animal was transferred with a platinum wire pick to a fresh newly seeded NGM plate and worm tracking was initiated at the moment that the animal’s image was captured, up to half a minute following transfer. All movies track the animals for 20 min.

### Cell Culture (HEK293T) and Transfection

Cell culture and transfection were performed as described (Geron et al., 2018). Briefly, human embryonic kidney 293T (HEK293T) cells were transfected with a total of 1 μg of DNA (GTL-1 pCNA4/TO alone or GTL-1 pCNA4/TO + hM3D(Gq) (a gift from Bryan Roth (Addgene plasmid # 45547; Gq/Dreadd)) and with pCDNA3.1(+) using Mirus LT1 transfection reagent (Mirus Bio, Madison, WI) according to manufacturer’s protocol. Co-transfection with EGFP in the pCDNA3.1(+) vector was carried for quick identification of successful transfection. Transfections were performed in 12-well plates containing 3x10^5^ cells 48h before analysis. Cells were plated on 0.05 mg/ml PDL-coated glass coverslips (12 mm) and incubated at 37°C (5% CO2) for at least 16h before electrophysiological analysis.

### Patch Clamp Recordings

Voltage-clamp recordings from transfected HEK293T cell were carried out as previously described (Kumar et al., 2017; Geron et al., 2018). Briefly, membrane currents were recorded under the voltage-clamp using an Axopatch 200B patch-clamp amplifier (Molecular Devices, Sunnyvale, CA, USA). Membrane currents were digitized using a Digidata 1440A interface board and pCLAMP 10.6 software (Molecular Devices, Sunnyvale, CA, USA) with sampling frequency set to 5 kHz and were low-pass filtered at 2 kHz. Holding voltage was −40 mV for transfected HEK293T. Patch electrodes were fabricated from borosilicate glass using the P1000 Micropipette Puller (Sutter Instrument) and fire-polished using the Microforge MF-900 (Narishige, Japan) to a resistance of 2–4 MΩ for both the inside-out and perforated patch recordings.

The inside-out configuration was performed as previously described (Kumar et al., 2017). Briefly, both the pipette solution and intracellular solutions constituted (mM): 150 NaCl, 2.8 KCl, 2 CaCl_2_, and 10 HEPES, adjusted to pH 7.4 with NaOH. Following the establishment of the excised inside-out multichannel patch, patches were continuously superfused with extracellular solutions with or without calcium *via* the ValveBank perfusion system (AutoMate Scientific, Berkeley, CA, USA).

The perforated-patch configuration was carried out as previously described (Priel and Silberberg, 2004; Kumar et al., 2017). Briefly, the pipette solution constituted (mM): 75 K_2_SO_4_, 55 KCl, 5 MgSO_4_, 10 HEPES, adjusted to pH 7.2 with KOH. Nystatin (Sigma, St. Louis, MO, USA) was used for patch perforation at a working concentration of 200 μM. To this end, it was dissolved in Dimethyl Sulfoxide (DMSO; Sigma, St. Louis, MO, USA) to obtain a 55 mM stock solution, which following 1 min ultra-sonication was diluted in pipette solution to obtain working solution. Nystatin solutions were freshly prepared in the dark every 2 h. Only cells with series resistance of ≤15 MΩ were used for analysis. The extracellular solution contained (mM): 140 NaCl, 2.8 KCl, 2 MgSO_4_, 1.8 CaCl_2_, 10 HEPES, 10 D-glucose, adjusted to pH 7.4 with NaOH (Ringer solution). Once the perforated-patch or the standard whole cell configuration was established, cells were continuously superfused with extracellular solutions. To avoid effects from pre-exposure of the recorded cells to the applied agonist, cells were exposed to them once, allowing the recording of a single cell from each coverslip.

### Statistical Analysis

Statistical analyses were performed using the Matlab statistical analysis toolbox and Prism 7 (GraphPad Software, La Jolla, CA, USA). Student’s t-test and ANOVA were used to determine statistical significance. Electrophysiological analysis was performed by using pClamp 10.6 software.

## Results

### GTL-1 Participates in the Response to Cold Temperatures

**Figure 1:**
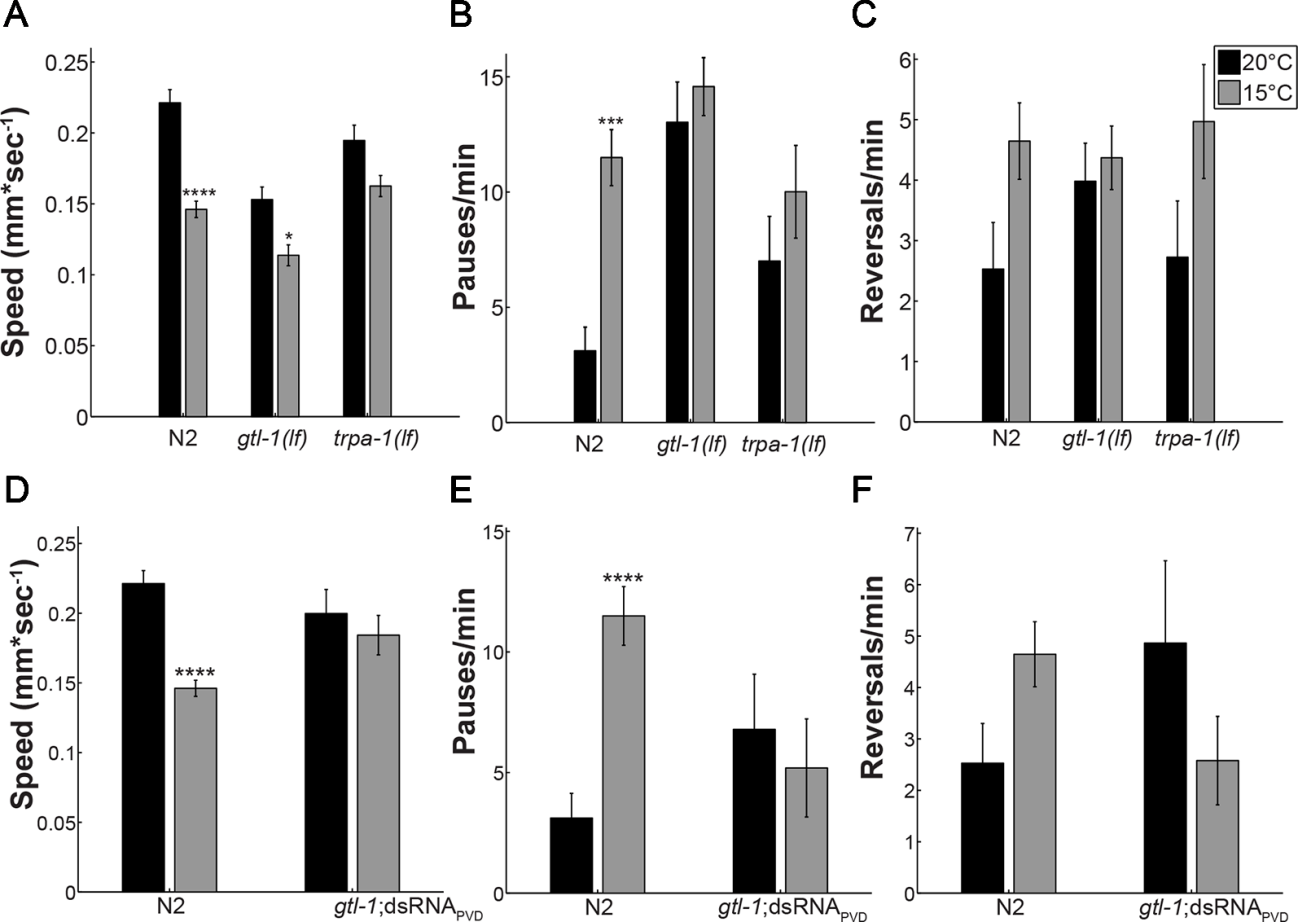
GTL-1 is required within PVD for the effects of temperature on locomotion. Effects of temperature on locomotion were compared between wild type (N2), *gtl-1(lf)*, and *trpa-1(lf)* animals **(A–C)** and between wild-type (N2) animals and animals expressing *gtl-1*dsRNA specifically in PVD (*gtl-1;*dsRNA_PVD_) **(D–F)**, at 20°C (black bars) or 15°C (grey bars). Number of animals, 24, 36, 29, 22, 18, and 15 **(A–C)** and 24, 36, 13, and 12 **(D–F)** in order of appearance. Significant differences for the same strain at 15°C relative to 20°C were examined using two-way ANOVA with bonferroni’s multiple comparisons correction (*-p < 0.05, ***-p < 0.01, ****-p < 0.0001). Temperature contributes significantly to the variance in A (p < 0.0001), B (p = 0.0018), C (p = 0.0112), D (p < 0.0001) and E (p = 0.042); genotype contributes significantly to the variance only in B (p < 0.0001); and effects of the interaction are significant in A (p = 0.0069), B (p = 0.0476), D (p = 0.0069), E (p = 0.0032), and F (0.026).

Previous analysis showed that PVD neurons respond to temperature downshift *via* the TRP channel, TRPA-1, as demonstrated by findings showing that *trpa-1(lf)* animals have an impaired response to rapid cooling. Specifically, in response to cooling *trpa-1(lf)* animals show no calcium transients within PVD and no increase in omega turns when examined in a liquid drop (Chatzigeorgiou et al., 2010). We have previously shown that TRPA-1 is required for the altered locomotion of animals at cold (15°C) relative to warm (20°C) temperatures (Cohen et al., 2014). To examine the role of GTL-1 in this behavioral response we used the same locomotion assay (Cohen et al., 2014). At 20°C, wild-type (N2) animals moved at an average speed of 0.225mm/sec and had a low rate of pauses and direction reversals (**Figure 1**). After 10 min at 15°C wild-type (N2) animals’ speed was significantly reduced and the rate of pauses was significantly increased relative to 20°C (**Figures 1A, B**, black bars vs. grey bars). In *gtl-1* mutants effects of temperature on speed were smaller, although significant; no significant effect of temperature on speed was detected in *trpa-1* mutants; and, no significant effect of temperature on the rate of pauses was seen in *gtl-1* and *trpa-1* loss of function mutants (**Figures 1A, B**). Temperature affected the rate of reversals similarly to its effect on the rate of pauses, but, in none of the strains was this effect significant (**Figure 1C**). The similar effects of GTL-1 and TRPA-1 in reducing the response to cold temperature relative to the response of wild-type animals are consistent with GTL-1, like TRPA-1, being required for this response (**Figures 1A, B**). We note that *gtl-1(lf)* also affects locomotion at 20°C; having significantly reduced speed and increased rate of pauses relative to wild-type (N2) animals (p < 0.0001 two-way ANOVA). However, contribution of genotype to the variance in locomotion is only significant when examining the rate of pauses (two-way ANOVA, p < 0.0001).

### Effects of GTL-1 on the Response to Cold Temperature Require Its Expression in PVD

Results in **Figures 1A–C** show altered locomotion of *gtl-1(lf)* animals at normal growth temperature (20°C). Such differences may mask the effect of temperature reduction on locomotion. Moreover, *gtl-1* is widely expressed and some of its effects may not depend on PVD expression. To examine whether the reduced response to cold temperatures depends on function of GTL-1 in PVD cells, we specifically knocked down its expression in PVD neurons using cell specific dsRNA-mediated knockdown (Esposito et al., 2007). Indeed, results in **Figures 1D, E** show that effects of GTL-1 on locomotion at 20°C do not depend on its expression in PVD. Moreover, genotype does not significantly contribute to the variance in locomotion when comparing wild-type animals to animals in which *gtl-1* was specifically silenced in PVD (two-way ANOVA). Importantly, effects of cold temperature on locomotion are eliminated in the transgenic animals in which *gtl-1* was specifically knocked-down in PVD. In addition, the residual effects of temperature on locomotion speed seen in *gtl-1(lf)* animals are not seen when *gtl-1* is specifically silenced in PVDs (compare **Figures 1A**–**D**). Thus, GTL-1’s function in PVD is required for the behavioral response of *C.elegans* to cold temperatures while effects of GTL-1 on locomotion under normal growth temperature are independent of PVD expression.

### Effects of GTL-1 on the Immediate Response to Cold

Results described above (**Figure 1**) show locomotion following adaptation to cold temperatures (10 min after transfer to 15°C) and may also include an enduring response to transfer, a strong mechanical stimulus (Cohen et al., 2012). To examine whether *gtl-1* participates in the immediate response to temperature downshift we used a setup that allows us to rapidly cool the agar surface on which the animals is placed while recording the animal’s locomotion (see Methods). Due to poor image quality in this setup movies were manually analyzed. Therefore, only one parameter was tested, the number of reversals. Reversals were counted during a 30 sec interval immediately before temperature downshift and immediately after temperature downshift and compared to the number of reversals in animals that were continuously maintained at 20°C. To reduce effects of transfer to the test surface on locomotion, animals were allowed to recover from the noxious mechanical stress (transfer with a wire pick) for 10 min before being examined.

**Figure 2:**
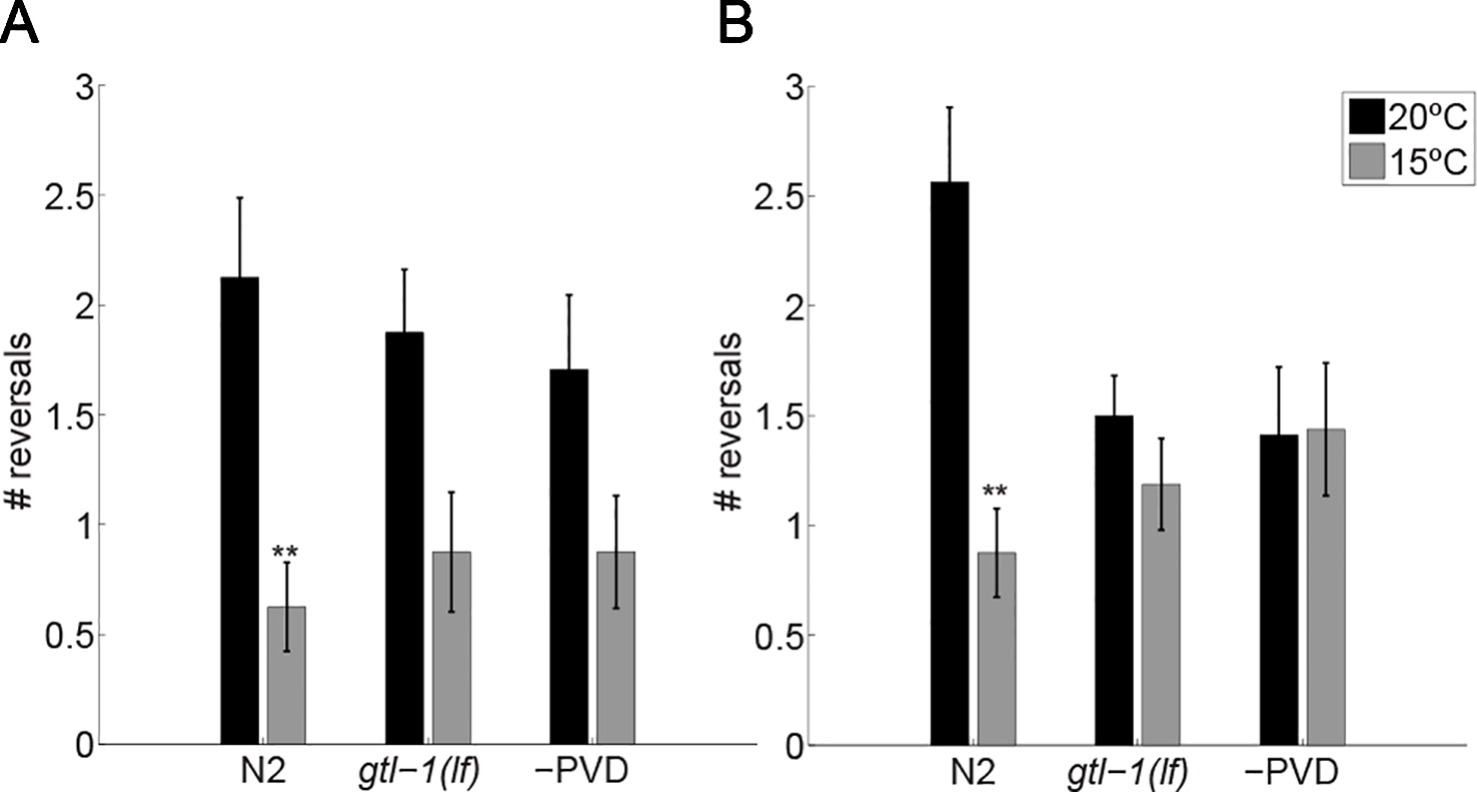
GTL-1 is required for the immediate and enduring response to cold temperatures. Number of reversals was counted over 30 sec immediately after the temperature drop **(A)** and 10 min later **(B)**. n = 16 each. Significant differences relative to controls, maintained at 20°C (black bars) throughout the experiments, were examined using two-way ANOVA **-p< 0.01).

After 10 min at 20°C, wild-type (N2) animals had a reversal rate of 4.25 ± 0.73 reversals/minute, when maintained at 20°C for another 10 min this rate slightly increased (5.25 ± 0.69 reversals/minute (**Figures 2A, B**, black bars)). Immediately after the temperature drop to 15°C wild-type animals reduced their rate of reversals (a 3.4-fold reduction to 1.25 ± 0.4 reversals/minute; **Figure 2A**), a reduction likely to represent an escape response i.e., increased forward speed, reduced pauses and reduced reversals (Cohen et al., 2012). This reduction is maintained for 10 min at the noxious temperature of 15°C (**Figure 2B**). Since, the response observed using this assay differs from the response to lower temperature seen in the previous assay [**Figure 1** and (Cohen et al., 2014)] we examined whether it represents a PVD-mediated response to temperature downshift. For this, we examined animals lacking PVD neurons [-PVD animals (Albeg et al., 2011)]. Indeed, -PVD animals exhibit a weaker, statistically insignificant reduction in reversals immediately after the temperature downshift (**Figure 2A**) and no reduction in the rate of reversals following 10 min at 15°C (**Figure 2B**). Thus, reduced rate of reversals following temperature downshift requires PVD neurons allowing us to use this assay to examine the role of GTL-1 in the immediate response to thermal stimuli. Indeed, this assay demonstrates the similarity between *gtl-1(lf)* and -PVD animals in their immediate and enduring responses to temperature downshift (**Figures 2A, B**). Together, our results (**Figures 1** and **2**) demonstrate an important role for GTL-1 in the response to cold temperatures.

### GTL-1 and the Response to Mechanical Stimuli

**Figure 3:**
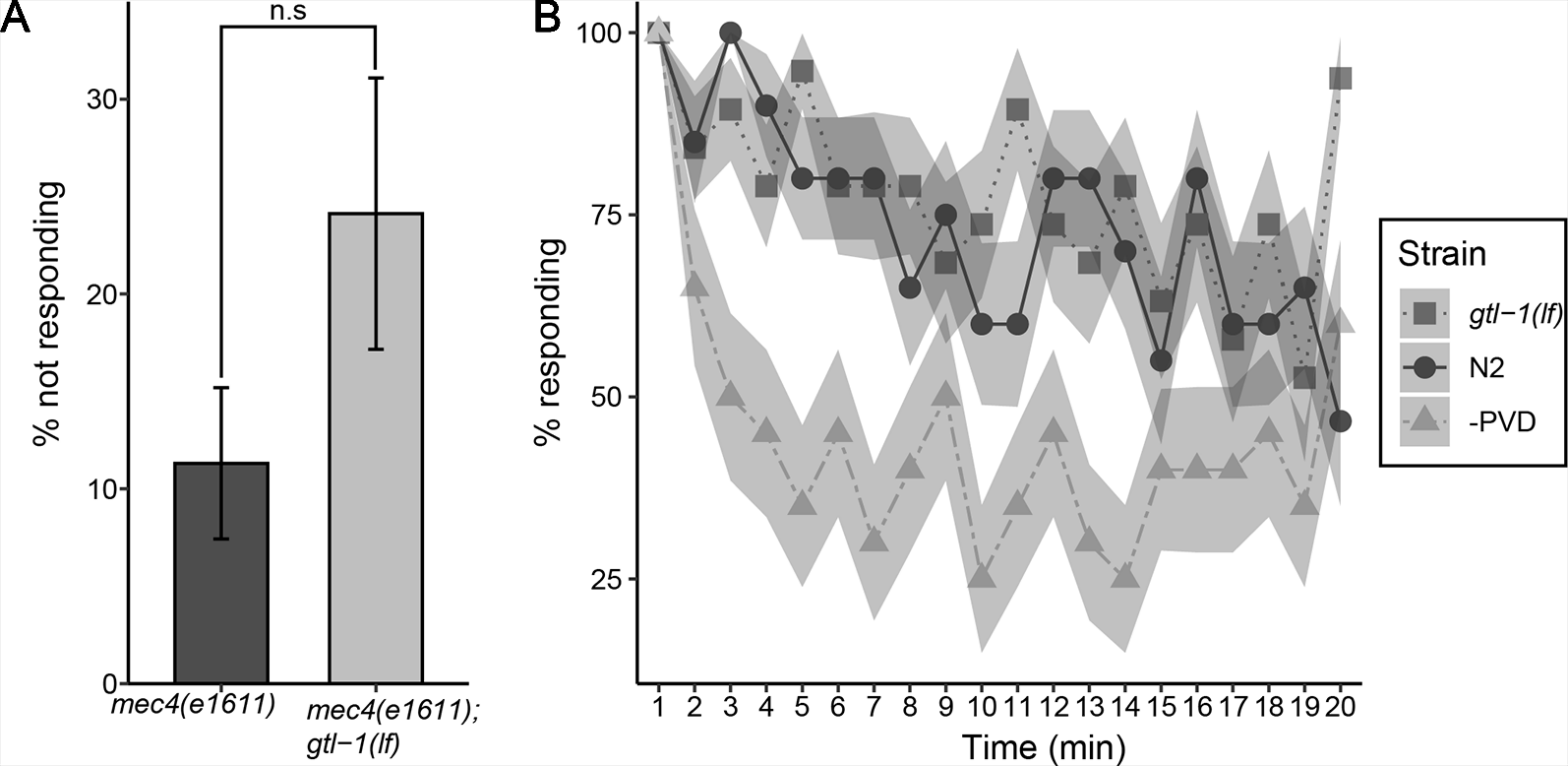
GTL-1 is not essential for the response to single or repeated mechanical stimuli. **(A)** Percent animals not responding to a single high threshold mechanical stimuli in *mec-4(e1611);*gtl-1(lf) and in *mec-4(1611)* animals (20 and 11 plates, respectively, 10 animals each plate). Statistical analysis was performed using n.s, p = 0.086. **(B)** Responses to repeated high threshold mechanical stimuli in *gtl-1(lf)* animals (n = 20), wild-type (N2, n = 20), and -PVD (n = 20) animals.

GTL-1 was shown to amplify optogenetic stimuli to PVD, a result suggesting that it functions downstream to nocisensors (Husson et al., 2012). Thus, GTL-1 is unlikely to function specifically in the response to thermal stimuli and is likely to also affect the response to noxious mechanical stimuli. To examine the role of GTL-1 in the response to mechanical stimuli we first examined its effects on percent animals responding to single noxious mechanical stimulus (prodding with a platinum wire pick). Since this response depends on both PVD and touch receptor neurons (Way and Chalfie, 1989) we compared responses of *mec-4(e1611)* animals, in which touch receptor neurons degenerate (Driscoll and Chalfie, 1991), to responses of *gtl-1(lf);mec-4(e1611)* lacking GTL-1 and touch receptor neurons. Using this assay we found a small but insignificant effect of GTL-1 on percent animals responding to noxious mechanical stimuli (**Figure 3A**). Thus *gtl-1* is not essential for a reliable response to a single high-threshold mechanical stimulus.

Responses to noxious stimuli habituate to a lower extent relative to responses to similar non noxious stimuli; as shown when comparing responses to repeated optogenetic activation of PVD neurons relative to responses to repeated optogenetic activation of the low threshold touch receptor neurons (Husson et al., 2012). To examine whether GTL-1 is required for maintaining the response to repeated mechanical stimuli we compared decay in percent animals responding to repeated noxious mechanical stimuli between wild-type, *gtl-1(lf)* and –PVD animals. Results of this analysis are consistent with PVD neurons being needed for maintaining the response to repeated mechanical stimuli as, animals lacking PVD neurons show a stronger decay in the response to repeated prodding with a platinum wire pick relative to wild type animals (**Figure 3B**). Our results, however, show that GTL-1 is not required for maintaining the response to repeated noxious mechanical stimuli as its responses to repeated prodding are indistinguishable from these of wild-type animals (**Figure 3B**).

**Figure 4:**
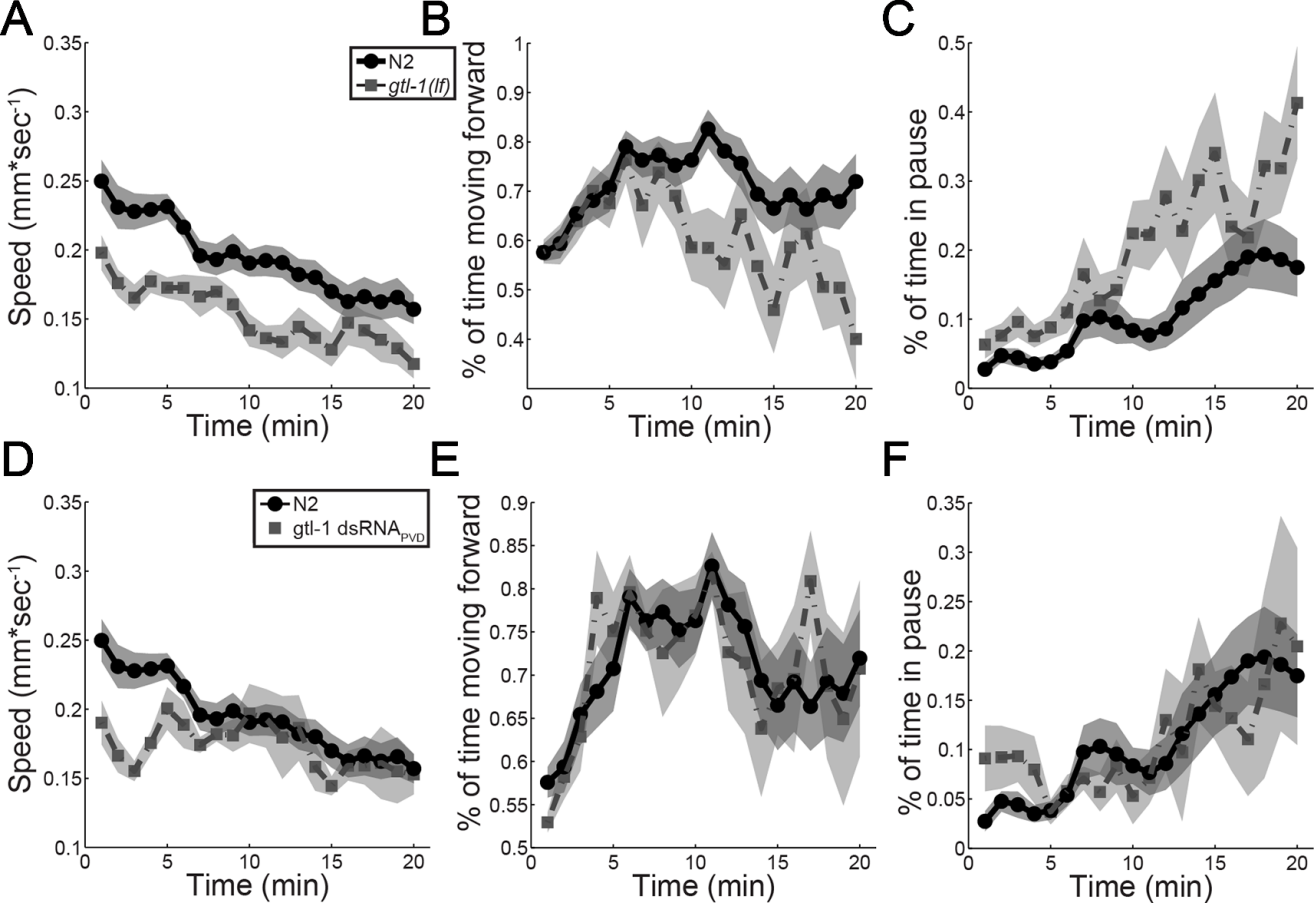
GTL-1 affects the magnitude of the response to noxious mechanical stimuli. Immediate and enduring responses to a noxious mechanical stimulus of wild-type (N2, N = 19, circles) compared to *gtl-1(lf)* (n = 13, squares) **(A–C)** and wild-type (N2, n = 19, circles) compared to *gtl-1* dsRNA_PVD_ (n = 5, squares) **(D–F)**.

Results in **Figure 3** show that GTL-1 is not required for producing a reliable behavioral response to high threshold mechanical stimuli or for maintaining a reliable response to repeated high threshold mechanical stimuli. However, these assays did not examine magnitude of the response to such stimuli. To examine whether GTL-1 affects response magnitude to mechanical stimuli, we imaged locomotion of animals for 20 min following transfer of the animal to a new plate with a platinum wire pick; this assay provides a sensitive and quantitative measure for roles of genes and neurons in the response to mechanical stimuli (Cohen et al., 2012). Results of this analysis show that both N2 and gtl-1(lf) animals show similar reduction of forward speed over time and a similar ratio between speed at the start point and the end point (1.59 and 1.68, respectively). However, the starting speed of gtl-1(lf) is lower then that of wild-type animals (**Figure 4A**). Analysis of two other locomotion parameters, percent time moving forward and percent time pausing (**Figures 4B, C**) show a similar starting point but larger changes over time relative to wild-type. To better understand the role of PVD expressed GTL-1 in locomotion following noxious mechanical stimuli we examined animals following PVD-specific knockdown of gtl-1 expression, as described above (**Figures 1D**–**F**). Results of this analysis show reduced initial speed following transfer of the animal with a platinum wire pick, a difference that is maintained 3 to 4 min following transfer (**Figure 4D**). Other parameters examined show no difference between wild-type animals and transgenic animals (**Figures 4E, F**). These results demonstrate a role for PVD-expressed GTL-1 in determining the magnitude of the escape response (enhanced forward speed) following a noxious mechanical stimulus. Other effects of GTL-1 on locomotion in this assay are likely to depend on its expression in yet unidentified cells. Previously published results show that animals lacking PVD show a similar reduction in the escape response, relative to wild-type animals, i.e. reduced speed immediately following transfer with a wire pick, a reduction that lasts for a few min (Cohen et al., 2012). Thus, GTL-1 is likely to be an important determinant of this PVD-mediated escape response to noxious mechanical stimuli.

### Heterologous Expression of GTL-1

Behavioral analysis (above) together with the previously published optogenetic analysis (Husson et al., 2012) show that GTL-1 functions in PVDs to enhance responses to multiple modalities. Previous studies suggest that GTL-1 might be a calcium-activated non-selective cation channel (CAN) (Xing et al., 2008; Xing and Strange, 2010), a mode of gating similar to that of TRPM4 and TRPM5 (Launay et al., 2002; Hofmann et al., 2003). Activation by cytosolic calcium may explain the role of GTL-1 in amplifying responses to multiple distinct stimuli activating PVDs.

**Figure 5:**
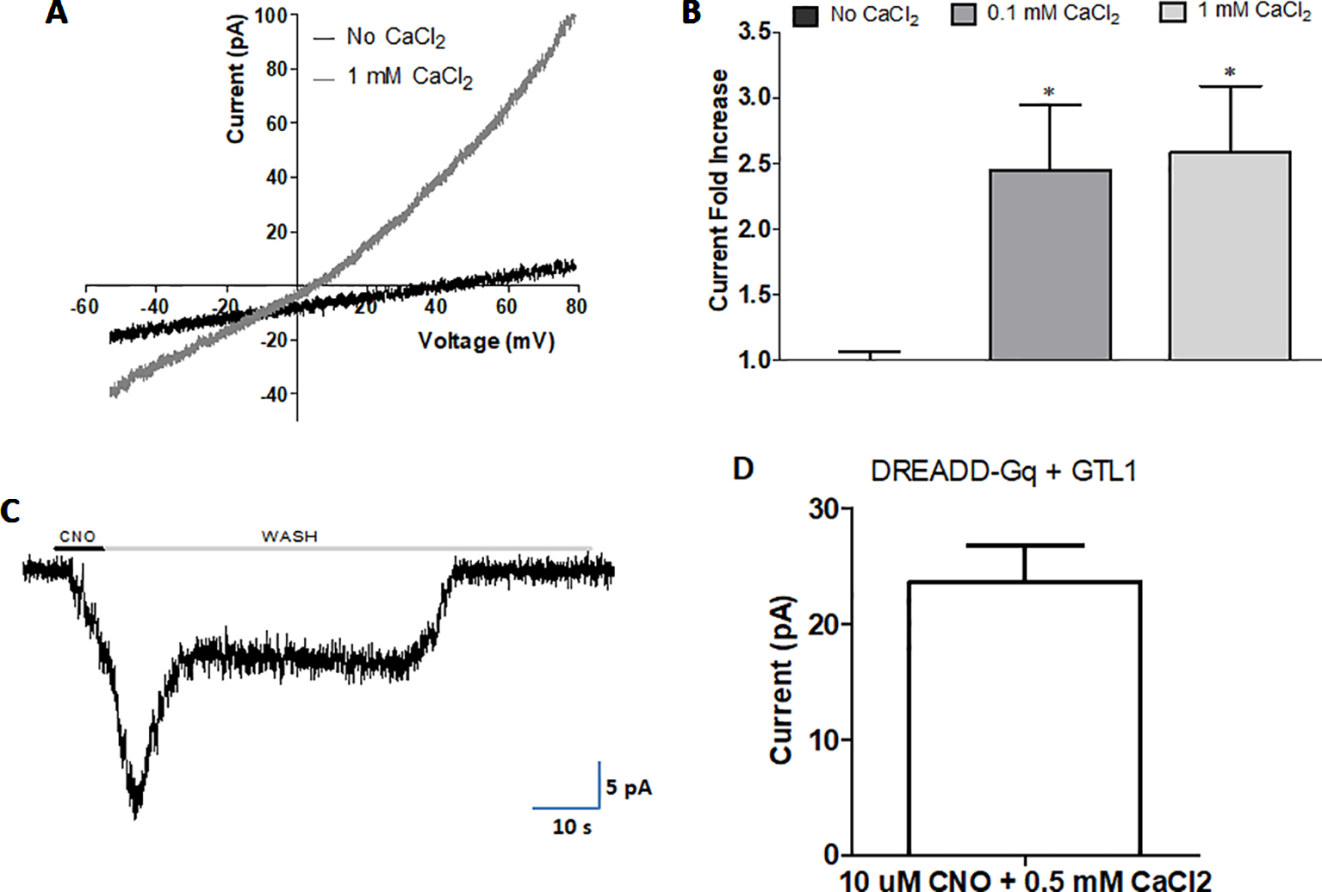
Internal calcium and Gαq activity gate GTL-1. GTL-1 was expressed in HEK293 cells. **(A and B)** Calcium enhances GTL-1 dependent currents. **(A)** Representative IV recording with and without calcium applied to the inner side of the excised inside-out patch. **(B)** Fold effect on current amplitudes of Calcium applied to the excised inside-out patch at +80mV. Effects of calcium are significant, p < 0.05, (n = 8), one-way ANOVA with correction for multiple variables **(C** and **D)** GTL-1 is activated by DREADD-Gαq. **(C)** Representative response. **(D)** Average response, n = 5. Of note, no currents are detected in the absence of CNO or in cells not expressing GTL-1.

**Figure 6:**
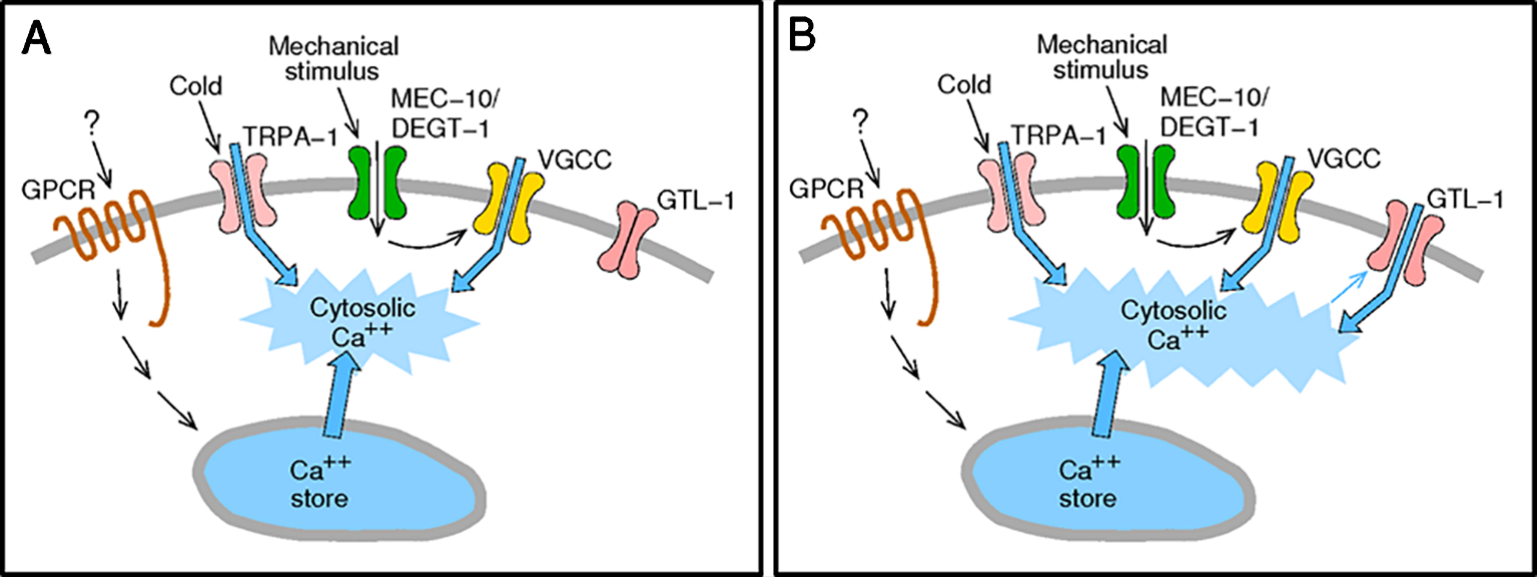
Activation of GTL-1 by internal calcium enables amplification of sensory stimuli. **(A)** Sensory stimuli *via* nocisensor activation directly (TRPA-1) or indirectly (GPCRs and MEC-10/DEGT-1) elevate cytosolic calcium. **(B)** Elevated cytosolic calcium activates GTL-1 channels to amplify the calcium signal.

To examine whether internal calcium activates GTL-1-dependent currents we used the multi-channel inside-out configuration of the patch clamp technique in HEK293 cells; excised patches were exposed to Ca^2+^ at different membrane voltages; this analysis showed voltage and internal calcium dependent gating of GTL-1 (**Figures 5A, B**). Specifically, in cells expressing GTL-1 and in the presence of internal calcium we observed outwardly rectifying currents (**Figure 5A**). Although, these currents were small they were significantly larger then currents seen in the absence of calcium (**Figure 5B**). No difference was observed between effects of 0.1 mM and 1 mM calcium on current amplitudes (**Figure 5B**); a result consistent with results obtained in the *C. elegans* intestine where GTL-1 and GON-2 function together to produce calcium oscillations and the defecation rhythm; these calcium oscillation were attributed to calcium having two opposing effects on these channels; activation at low concentrations and inhibition at higher concentrations (Estevez and Strange, 2005). Importantly, activation of GTL-1 by internal calcium enables its activation by receptors and channels affecting cytosolic calcium levels, directly or indirectly (**Figure 6**).

In the intestine GTL-1 was suggested to function downstream to PLC and IP_3_, known to function as part of signaling pathways leading to calcium release from internal stores (Xing and Strange, 2010). To examine whether G_α_q-dependent signaling, leading to PLC activation and IP_3_ signaling, activates GTL-1 we used a Designer Receptor Exclusively Activated by Designer Drugs (DREADD) developed for expression in HEK293 cells (Lee et al., 2014; Roth, 2016). Previously published results show that in HEK293 cells CNO-dependent currents are only detected when this DREADD receptor is co-expressed with a TRP channel and are abolished in the presence of a PLC inhibitor (Kumar et al., 2017). Thus, results (**Figures 5C, D**) showing reproducible CNO-activated currents when co-expressing GTL-1 with this DREADD receptor are a clear indication for GTL-1 being activated downstream to G_α_q-dependent signaling and are consistent with results obtained in the *C. elegans* intestine (Xing and Strange, 2010).

## Discussion

GTL-1, a member of the TRPM family of ion channels, was previously shown to amplify behavioral responses to optogenetic activation of PVD (Husson et al., 2012). Here, we set out to characterize the role of GTL-1in PVD-mediated responses to noxious, physiological, stimuli. PVD cells mediate the response to temperature downshift, a response requiring PVD expression of TRPA-1 (Chatzigeorgiou et al., 2010). Our results demonstrate similar defects in the behavioral responses to cold temperatures in *gtl-1(lf)* animals, *trpa-1(lf)* animals, and animals lacking PVD. Analysis of the PVD mediated response to mechanical stimuli, however, shows milder GTL-1-dependent behavioral effects. Specifically our results show reduced response magnitude to a noxious mechanical stimulus. But, no significant reduction in the reliability of the response (percent animal responding) to a single or to repeated mechanical stimuli. To understand the mechanism enabling effects of GTL-1 on responses to thermal, mechanical, and optogenetic stimuli, we examined its gating using electrophysiology in HEK293 cells. Results of this analysis show that internal calcium and Gαq-signaling gate this channel. Thus, GTL-1 like other TRPM channels, and as described in **Figure 6**, enables amplification of responses to receptors and channels that, directly or indirectly, enhance internal calcium levels (Launay et al., 2002; Zurborg et al., 2007).

Our results suggest that GTL-1 is mainly required for responses to immediate and enduring cold temperatures while having a relatively minor role in the response to high threshold mechanical stimuli. Non-sensor channels that are required for a specific modality within a polymodal nociceptor have previously been reported. For example, inhibition of the voltage-gated potassium channel, k_v_1.1, leads to severe mechanical allodynia (i.e., lowers the threshold and causes non-painful stimuli to be sensed as painful) without affecting the threshold for noxious heat (Hao et al., 2013); the voltage-gated sodium channel, Na_v_1.8, on the other hand, has been shown to specifically affect transmission of cold sensation, by being the only voltage-gated sodium channel active at low temperatures (Matthews et al., 2006; Zimmermann et al., 2007). Additional analysis of GTL-1 channel’s properties is needed to better understand its modality specific effects.

In mammals, the TRPM family consists of 8 different channels TRPM1-8. Members of this family are implicated in various biological processes, e.g., regulation of Ca^2+^ oscillations following T-lymphocyte activation (TRPM4) (Launay et al., 2004), regulation of magnesium absorption in kidneys and the intestine (TRPM6) (Voets et al., 2004; Vriens et al., 2011), gustatory transduction (TRPM5) and innocuous and noxious thermal sensing (TRPM3 and 8)(Zhang et al., 2003; Bautista et al., 2007; Dhaka et al., 2007; Vriens et al., 2011). *C. elegans* expresses three TRPM channels, *gon-2, gtl-1*, and *gtl-2.* These channels express widely including the intestine, the gonad, excretory cells, and the nervous system. Functions of these channels include, maintaining Ca^2+^ and Mg^2+^ homeostasis in the intestines and in extracellular fluids (Teramoto et al., 2005; Stawicki et al., 2011), controlling defecation rhythm (Kwan et al., 2008; Xing et al., 2008), and regulating the onset and continuation of post-embryonic mitotic cell divisions in the somatic gonad (Sun and Lambie, 1997; West et al., 2001). Our results combined with the results in (Husson et al., 2012) show for the first time a neuronal function for GTL-1; enhancing responses to noxious stimuli.

Previous work showed that in the *C. elegans* intestine GTL-1 functions together with GON-2 to produce a strong inwardly rectifying current, I_ORCa_. The role of GTL-1 in this current was deduced from comparing currents in *gtl-1, gon-2*, or *gon-2;gtl-1* loss of function animals to wild-type animals. These studies suggested that GTL-1 has a minor role in I_ORCa_ and that it is likely to be activated by cytosolic Ca^2+^ and to function downstream of PLC signaling (Teramoto et al., 2005; Xing et al., 2008; Xing and Strange, 2010). This study, for the first time, characterizes properties of the GTL-1 channel in a heterologous expression system. Results of this analysis are consistent with the *in-vivo* results, showing mild outward rectification of the GTL-1 dependent currents, activation by internal calcium and by Gαq signaling. Importantly, our findings suggest a mechanism, summarized in **Figure 6**, whereby GTL-1 is activated downstream of channels, receptors and signaling pathways whose activation directly or indirectly increases cytosolic calcium; thus enabling GTL-1 dependent enhancement of multiple and distinct sensory modalities as demonstrated by its effects on behavioral responses to thermal and mechanical stimuli.

## Data Availability Statement

The datasets generated for this study are available on request to the corresponding author.

## Author Contributions

EC performed behavioural analysis, cloned *gtl-1* cDNA into mammalian cell expression vector, organized results for article, and participated in writing the article. RK performed and analyzed heterologous expression studies. TZ set up the system enabling imaging and analysis of the response to rapid temperature downshift, performed the experiments, and analyzed them. AP supervised the heterologous expression studies and participated in writing the article. MT supervised behavioral analysis and wrote the article.

## Funding

This work was supported by the Prusiner-Abramsky research award to MT, the Israel Science Foundation (Grants 1444/16 to AP), the Brettler Center and David R. Bloom Center, School of Pharmacy (The Hebrew University of Jerusalem; to AP), a Jerusalem Brain Committee Postdoctoral Fellowship (to RK).

## Conflict of Interest

The authors declare that the research was conducted in the absence of any commercial or financial relationships that could be construed as a potential conflict of interest.
